# Genetic Correlations and Causal Relationships Among Allergic Diseases: A Comprehensive Mendelian Randomization Study With Multiomic Mediation Analysis

**DOI:** 10.1155/mi/5466012

**Published:** 2026-04-09

**Authors:** Ping-An Zhang, Jie-Lin Wang, Shi-Yan Fu, Hua-Lian Luo, Nai-Jian Li, Run-Dong Qin, Jing Li

**Affiliations:** ^1^ State Key Laboratory of Respiratory Disease, National Clinical Research Center for Respiratory Disease, Guangzhou Institute of Respiratory Health, Department of Allergy and Clinical Immunology, The First Affiliated Hospital of Guangzhou Medical University, Guangzhou, Guangdong, China, gzhmc.edu.cn; ^2^ Department of Obstetrics and Gynecology, Department of Gynecologic Oncology Research Office, Guangzhou Key Laboratory of Targeted Therapy for Gynecologic Oncology, Guangdong Provincial Key Laboratory of Major Obstetric Diseases, Guangdong Provincial Clinical Research Center for Obstetrics and Gynecology, Guangdong-Hong Kong-Macao Greater Bay Area Higher Education Joint Laboratory of Maternal-Fetal Medicine, The Third Affiliated Hospital, Guangzhou Medical University, Guangzhou, China, gzhmu.edu.cn

**Keywords:** allergic diseases, inflammatory mediators, Mendelian randomization, multiomics, serum proteomics, type 2 inflammation

## Abstract

**Background:**

Allergic diseases, including allergic asthma (AA), allergic rhinitis (AR), atopic dermatitis (AD), and allergic conjunctivitis (AC), often coexist. However, the specific inflammatory mediators driving their shared mechanisms remain unclear. This study explored causal relationships and identified multiomic mediators among allergic diseases using Mendelian randomization (MR).

**Methods:**

Large‐scale GWAS datasets from FinnGen and UK Biobank were analyzed. Linkage disequilibrium score regression (LDSC) and MR–robust adjusted profile scoring (MR‐RAPS) assessed genetic correlations and causal links. Crucially, multivariable MR (MVMR) adjusted for classical type 2 inflammatory factors to isolate independent effects. A two‐step MR framework evaluated the mediating roles of immune cells, metabolites, gut microbiota, and serum proteins.

**Results:**

Six disease pairs (AA–AR, AA–AD, AR–AA, AR–AD, AD–AA, and AD–AR) showed robust causal relationships independent of type 2 inflammation markers. Mediation analyses identified key inflammatory mediators, including immune cell subsets (e.g., CD45 on granulocytes), plasma metabolites (e.g., pentose acid), gut microbiota (e.g., *Bacteroides intestinalis*), and notably, serum proteins such as ABHD12 and SEZ6L2. Pathway analyses highlighted cytokine–cytokine receptor interactions and lipid metabolism.

**Conclusions:**

This study maps a comprehensive causal network of allergic multimorbidity. We identified novel serum proteins and metabolic mediators beyond the classical type 2 inflammation axis, offering potential therapeutic targets for disrupting the inflammatory crosstalk between allergic diseases.

## 1. Introduction

Allergic diseases are systemic disorders caused by immune system dysregulation, including allergic asthma (AA), atopic dermatitis (AD), allergic rhinitis (AR), and allergic conjunctivitis (AC). The prevalence of allergic diseases has been steadily increasing in recent years, becoming a major global public health concern [1]. According to the latest estimates from the Global Burden of Disease study, there were ~260 million asthma patients and 129 million AD patients worldwide in 2021, with the total number of cases projected to continue rising by 2050 [2]. The prevalence of AR in the European population is estimated to range from 17% to 29% [3], and its incidence has steadily increased in recent years [4, 5]. AC is highly prevalent, affecting 10% to 20% of people globally [6, 7]. Due to their chronic and recurrent nature, these diseases significantly impair quality of life as well as mental health. Furthermore, the costs of diagnosis, treatment, and management place a heavy economic burden on individuals, families, and healthcare systems [8, 9].

Clinical and epidemiological studies indicate that asthma, AR, AC, and AD often co‐occur. Notably, recent meta‐analytic evidence encompassing over five million subjects indicates that AR and AC exhibit strong associations with asthma (odds ratios of 4.24 and 2.63, respectively) [10]. Specifically, an observational study reported that moderate to severe (M/S) asthma patients have a high prevalence of comorbid AR (60%) and AD (15%) [11]. Allergic diseases often coexist and significantly contribute to the overexpression of type 2 inflammatory pathways, involving the activation of type 2 helper T cells and type 2 innate lymphoid cells [12]. However, emerging evidence suggests that other pathways, including Th17‐mediated inflammation, regulatory T (Treg) cell dysfunction [13, 14], and nonimmune factors, may also contribute.

Allergic diseases are systemic disorders associated with the body’s internal homeostasis, involving complex multifactorial mechanisms such as immune responses, metabolism, and gut microbiota. The complex interplay between inflammation and immune responses lies at the core of the pathophysiology of allergic diseases [15]. This dynamic interaction involves the activation of various immune cells, release of cytokines, and recruitment of inflammatory mediators, which together drive the chronic and often relapsing nature of these conditions [16]. Emerging evidence indicates that metabolic dysregulation is associated with allergic diseases [17]. Short‐chain fatty acids (SCFAs) act as key molecules in the pathogenesis and progression of allergic conditions by engaging specific receptors and inhibiting histone deacetylases [18]. Deprivation of exogenous proline disrupts metabolic reprogramming and inhibits the AKT/mTORC1 and WNT3a/β‐catenin signaling pathways, thereby suppressing house dust mite–induced EMT and alleviating asthma [19]. Additionally, the gut microbiota, as a key regulator of immune function, has been recognized for its pivotal role in the development and progression of allergic diseases by influencing immune responses and metabolic balance [20, 21]. A deeper understanding of the immune system, metabolites, and gut microbiota will enhance comprehensive insights into the systemic effects and interactions of allergic diseases and provide novel avenues for precision therapy.

Previous studies have demonstrated phenotypic transitions among allergic diseases, with the majority of research emphasizing type 2 inflammation as the primary pathogenic mechanism [22–24]. However, emerging evidence highlights the role of genetic susceptibility [25] alongside immune dysregulation [26], metabolic disturbances [27], and gut microbiota alterations [28] in the etiology and progression of these conditions. Accordingly, we hypothesize that significant genetic correlations exist among allergic diseases and that their phenotypic conversions are closely linked to complex interactions involving immune, metabolic, and microbiome factors. To date, most studies have primarily focused on how inflammation, immunity, and metabolism influence allergic diseases [29, 30], with relatively little attention paid to how allergic diseases themselves trigger systemic homeostatic changes. Furthermore, many observational studies have employed case–control designs, making it difficult to establish the temporal sequence between exposures and disease outcomes, as well as to infer causality. Mendelian randomization (MR) is a reliable epidemiological technique that uses genetic variants associated with a risk factor to establish a cause‐and‐effect relationship between exposure and outcome. In this study, we first explore the genetic correlations among allergic diseases using linkage disequilibrium score regression (LDSC). We then investigate causal relationships between allergic diseases through MR–robust adjusted profile scoring (MR‐RAPS) analyses. To demonstrate that causal relationships among allergic diseases persist even after adjusting for type 2 inflammation–related factors (such as eosinophils and IgE), we employ multivariable MR (MVMR). Building on this, we further explore potential mediators beyond type 2 inflammation that may contribute to the transition between allergic diseases by applying a two‐step, two‐sample MR approach.

## 2. Method

### 2.1. Study Design

Large‐scale GWAS datasets from the FinnGen and UK Biobank cohorts were utilized in the study.

First, LDSC was employed to assess genetic correlations, revealing significant genetic associations among four pairs of allergic diseases (AA–AD, AA–AR, AR–AD, and AR–AC).

Second, to systematically explore causal relationships, a series of bidirectional two‐sample MR analyses was conducted. In this framework, the four pairs of genetically correlated allergic diseases identified in the LDSC analysis were sequentially treated as potential exposures, with the corresponding diseases considered as outcomes, yielding a total of eight pairs. This bidirectional design is essential for disentangling the potentially reciprocal relationships and feedback loops among these highly comorbid conditions. To uncover potential causal links among the four allergic diseases, MR‐RAPS was applied to identify causal associations, and the findings were validated in an independent cohort. Ultimately, six pairs of allergic diseases demonstrated significant causal relationships.

Third, MVMR, adjusting for type 2 inflammation–related markers such as eosinophil counts, IgE, IL‐4, IL‐5, and IL‐13, was used to examine the causal relationships among allergic diseases.

**Figure 1:**
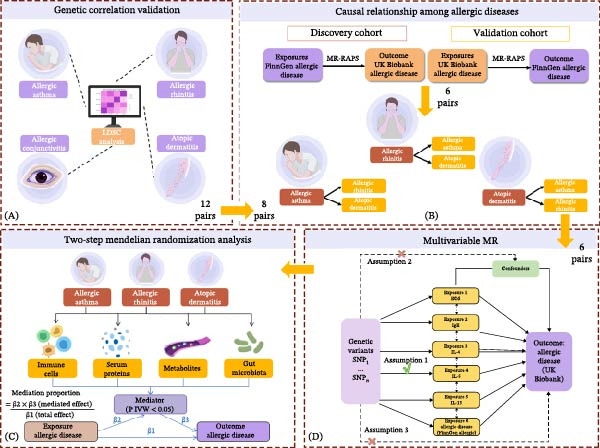
Overview of study design and analysis pipeline. (A) Genetic correlation analysis by LDSC of four allergic traits (AA, AR, AD, and AC) using FinnGen and UK Biobank GWAS; 12 disease pairs showed significant genetic correlation. (B) Bidirectional causal inference by MR‐RAPS in discovery and validation cohorts; six disease pairs confirmed. (C) Multivariable MR of the six confirmed pairs, adjusting for eosinophil count, IgE, IL‐4, IL‐5, and IL‐13. (D) Two‐step MR mediation: For each of the six causal disease pairs, two‐sample MR was conducted to screen 731 immune cell traits, 871 plasma metabolites, 473 gut microbial taxa, and 4782 circulating proteins as potential mediators.

Finally, two‐step MR analysis further explored the mediation effects of immune cells, plasma metabolites, gut microbiota, and serum proteins, while functional enrichment and network analyses were conducted to elucidate the underlying biological mechanisms. The overall workflow is illustrated in Figure 1.

MR is based on three fundamental assumptions: firstly, instrumental variables (IVs) have a significant correlation with exposure factors; secondly, IVs are not connected to any confounding factors; and thirdly, IVs are only associated with outcomes through exposure. These assumptions improve the dependability of the argument for associations in MR [31]. We followed the guidelines provided in the Strengthening the Reporting of Observational Studies in Epidemiology‐MR (STROBE‐MR) checklist for our study [32] (Supporting Information 1).

### 2.2. Data Source

We obtained GWAS datasets for four allergic diseases—AA, AR, atopic conjunctivitis, and AD—from both the FinnGen and UK Biobank databases. Table 1 summarizes these five allergic disease datasets.

**Table 1:** Summary of allergic disease datasets.

Disease	Data source	Sample size	*N* control	*N* case	GWAS ID
Allergic asthma	FinnGen	283,740	270,290	13,450	finngen_R12_ALLERG_ASTHMA
	UK Biobank	462,013	408,756	53,257	ukb‐b‐20296
Allergic rhinitis	FinnGen	490,219	474,650	15,569	finngen_R12_ALLERG_RHINITIS
	UK Biobank	462,933	436,826	26,107	ukb‐b‐16499
Atopic conjunctivitis	FinnGen	500,348	470,557	29,791	finngen_R12_H7_ALLERGICCONJUNCTIVITIS
	UK Biobank	361,194	—	—	—
Atopic dermatitis	FinnGen	464,119	432,874	31,245	finngen_R12_L12_ATOPIC
	UK Biobank	462,933	451,114	11,819	ukb‐b‐20141

The metabolite dataset was sourced from Chen et al. [33], who analyzed 1091 blood metabolites and 309 metabolite ratios across 8299 individuals, covering 150,000 SNPs. From these, we retained 871 metabolites after excluding unknown compounds and all ratio measures. Meanwhile, the gut microbiota dataset was acquired from Qin et al. [34], who conducted comprehensive genome‐wide analyses of 473 gut microbiota species, with data available in the NHGRI‐EBI GWAS database (accession numbers GCST90032172 to GCST90032644). We obtained serum proteins GWAS datasets from Vilmundur et al., who conducted an extensive genome‐wide association study on 4782 serum proteins encoded by 4135 unique human genes in the population‐based AGES cohort of elderly Icelanders. Protein measurements were performed using the slow‐off rate modified aptamer (SOMAmer) platform. The datasets are available in the GWAS database under accession codes GCST90086175 to GCST90090956 [35]. Finally, immune cell trait datasets were derived from the work of Orrù et al. [36], who extensively analyzed 731 immune cell phenotypes and their immunological functions. Their study incorporated 539 experimental assays examining ~22 million genetic variants in 3757 Sardinian participants. The corresponding GWAS datasets are accessible via accession codes GCST90001391 to GCST90002121 [36].

Additionally, the GWAS dataset for eosinophil cell count used in the MVMR analysis was obtained from the Blood Cell Consortium (Dataset: ieu‐b‐33) and comprises 563,946 individuals of European ancestry. The GWAS summary statistics for IgE levels were sourced from the study by Scepanovic et al. [37]. GWAS summary statistics for IL‐4, IL‐5, and IL‐13 were obtained from the large‐scale meta‐analysis of 40 circulating cytokines conducted by Konieczny et al. [38] in 74,783 individuals. Data on IL‐25 and IL‐33 were extracted from the GWAS meta‐analysis by Gudjonsson et al. [35], encompassing 2091 serum proteins measured in 5368 individuals. GWAS summary statistics for TSLP were obtained from the meta‐analysis conducted by Zhao et al. [39], which analyzed 91 serum proteins in 14,824 individuals.

### 2.3. Selection of IVs

In this study, we applied a rigorous multistep process to select valid IVs. First, we identified SNPs associated with allergic diseases using a genome‐wide significance threshold of *p*  < 5 × 10^−8^ [40]. For SNPs related to immune cells, serum proteins, metabolites, and the gut microbiome—where the *p*  < 5 × 10^−8^ cutoff would exclude most variants—we relaxed the threshold to *p*  < 5 × 10^−6^ to retain a sufficient number of candidates. Next, we used the “clump data” function to prune SNPs in linkage disequilibrium (LD), applying an *r* ^2^ < 0.001 criterion within a 10 000 kb window to ensure independence and remove nonbiallelic and LD‐linked variants. We then systematically removed palindromic variants to prevent strand ambiguity.

Finally, we systematically identified and excluded SNPs associated with known confounders such as smoking and body mass index (BMI). To achieve this, these SNPs were screened using the GWAS Catalog database (https://www.ebi.ac.uk/gwas/home) and removed prior to the final IV selection. Any variants showing a genome‐wide significant association (*p*  < 5 × 10^−8^) with these confounding traits were strictly removed prior to the final IV selection. This rigorous screening ensured that our IVs robustly and specifically reflect the exposure–outcome relationship.

### 2.4. Statistical Analysis

#### 2.4.1. Genetic Correlation Validation

We used LDSC to investigate the genetic correlations (rg) among four allergic diseases. LD scores for each SNP were computed to quantify its aggregate LD with neighboring variants [41]. We restricted our analysis to HapMap3 SNPs with minor allele frequency ≥ 0.01, discarding all others. Genetic correlations are reported as rg ± standard error (SE). If either trait exhibits low heritability, LDSC may not yield a valid estimate. Associations with *p*  < 0.05 were deemed suggestive of a shared genetic basis.

#### 2.4.2. MR‐RAPS

To uncover potential causal links among the four allergic diseases, we applied MR‐RAPS [42]. For these analyses, the four pairs of genetically correlated allergic diseases identified in the LDSC analysis were systematically tested for causal effects in both directions for each pair. Many allergy risk genes concurrently regulate multiple immune and inflammatory pathways, acting independently across different tissues or cell types, and thereby cause a single genetic variant to influence diverse allergic phenotypes. Given this high potential for widespread horizontal pleiotropy, which our prior work confirmed [43], traditional TSMR methods are often insufficient as their assumptions are violated. We therefore selected MR‐RAPS as our primary method for the disease‐to‐disease analyses. MR‐RAPS is specifically designed to provide robust causal estimates in the presence of such pleiotropy by using a robust weighting scheme; furthermore, unlike methods such as MR‐Egger, it is also robust to potential weak instrument bias, which enhances the reliability of the analysis [42].

We applied this MR‐RAPS analysis only to the disease pairs that showed significant genetic correlation in the LDSC analysis. To ensure the robustness of our causal findings, we employed a stringent reciprocal replication design. First, we conducted a “discovery” analysis using allergic disease phenotypes from FinnGen as exposures and matching UKB phenotypes as outcomes. Second, we performed a “validation” analysis by swapping the roles, using UKB phenotypes as exposures and FinnGen phenotypes as outcomes. A causal relationship was considered significant only after applying a Bonferroni correction for the multiple comparisons tested. Finally, only those disease pairs that demonstrated a significant causal relationship in both the “discovery” and “validation” analyses were retained for the subsequent exploratory mediation analyses.

#### 2.4.3. MVMR

To determine whether allergic diseases are causally interrelated, independent of type 2 inflammation, we applied MVMR [44]. MVMR augments the traditional MR framework by integrating genetic variants associated with multiple exposures, enabling the estimation of each exposure’s independent effect on the outcome while attenuating confounding bias. In this study, we conducted a two‐sample MVMR analysis of allergic diseases under a highly adjusted model. To minimize potential confounding, we adjusted for a comprehensive set of eight type 2 inflammation markers, including downstream mediators (eosinophil cell count, IgE, IL‐4, IL‐5, and IL‐13) and key upstream epithelial‐derived alarmins (TSLP, IL‐33, and IL‐25), for which we obtained corresponding GWAS summary statistics. Furthermore, to account for the mutual influence among the three major allergic phenotypes (AA, AR, and AD), we additionally included the third allergic disease as a covariate when assessing the causal effect between the other two.

#### 2.4.4. Two‐Step MR Analysis and Mediation Analysis

A two‐step MR framework was implemented to investigate potential mediators of the causal relationships among allergic diseases. Consistent with recent guidelines emphasizing methodological rigor and the use of multimethod approaches to address pleiotropy, stringent validation criteria were applied to all candidate mediators [45, 46].

In Step 1, MR‐RAPS—as described above—was used to confirm causal links between disease pairs. In Step 2, we conducted two‐sample MR to evaluate candidate mediators—immune cell traits, plasma metabolites, gut microbiota, and serum proteins. For each exposure–mediator–outcome triad, we required directional consistency (β_1_, the effect of exposure on outcome; β_2_, the effect of exposure on mediator; β_3_, the effect of mediator on outcome: when β_1_ is positive, β_2_ and β_3_ must share the same sign; when β_1_ is negative, they must have opposite signs) before calculating the indirect effect as β_2_ × β_3_ and the proportion mediated as (β_2_ × β_3_)/β_1_ [44, 47]. Crucially, to validate the unidirectional causal assumption required for mediation, a rigorous filtering step was implemented. Extensive bidirectional MR analyses were performed to test for reverse causality (i.e., from outcome to mediator and from mediator to exposure). Only mediators showing no significant evidence of reverse causal effects in these validation tests were retained for the final mediation analysis. Full results of the bidirectional tests are provided in Supporting Information Table S21. For the retained mediators, we applied multiple MR methods, including IVW (primary), MR‐Egger, weighted median, Wald ratio (for single‐SNP instruments), simple mode, and weighted mode [48]. A mediation effect was considered significant if it met two criteria: (1) the IVW *p*‐value was below 0.05 in the absence of heterogeneity or horizontal pleiotropy, and (2) the direction of the effect estimate was consistent across all applied MR methods.

#### 2.4.5. Sensitivity Analysis

To rigorously test the key MR assumptions, particularly potential violations by horizontal pleiotropy, we ensured robustness by performing comprehensive sensitivity analyses. Heterogeneity was assessed using Cochran’s *Q* test, where a *p*‐value greater than 0.05 indicated the absence of heterogeneity [49]. To assess horizontal pleiotropy, we employed MR‐Egger and MR‐PRESSO analyses; a *p*‐value greater than 0.05 suggested no evidence of horizontal pleiotropy. The MR‐PRESSO method was used to detect and remove significant outliers, thus minimizing the impact of horizontal pleiotropy [40, 50]. Additionally, we carried out a leave‐one‐out study, systematically excluding each SNP to determine whether a single SNP disproportionately affected the results [51].

### 2.5. Enrichment Analysis and Network Analysis

KEGG and GO pathway enrichment analyses were performed on the serum proteins (IVW *p*‐value < 0.05). Cytoscape (http://www.cytoscape.org) was used to visualize the protein–protein interaction (PPI) network [52], and core genes were identified in Cytoscape using the cytoHubba plugin with the Maximal Clique Centrality (MCC) algorithm. Transcription factor (TF) interaction prediction and miRNA–gene interaction analysis were performed using the NetworkAnalyst platform (https://www.networkanalyst.ca/) [53].

## 3. Result

### 3.1. Genetic Correlation Validation

We employed LDSC to assess genetic correlations between pairs of allergic diseases drawn from two independent cohorts (FinnGen and UK Biobank). In each analysis, one disease was taken from FinnGen and the other from UKB. We found that all examined disease pairs exhibit positive genetic correlations (Figure 2A). Genetic correlation was deemed significant when both directional *p*‐values were below 0.05. As detailed in Supporting Information Table S1, all disease pairs except AA–AC and AD–AC exhibited significant genetic overlap (Figure 2B). Overall, the majority of allergic disease combinations showed positive genetic correlations.

Figure 2Genetic correlation analysis across allergic diseases. (A) Heatmap showing linkage disequilibrium score regression (LDSC)–derived genetic correlation coefficients (rg) between each FinnGen and UK Biobank trait pair. (B) Forest plots displaying rg estimates with 95% confidence intervals for the same trait pairs.

**Figure:**
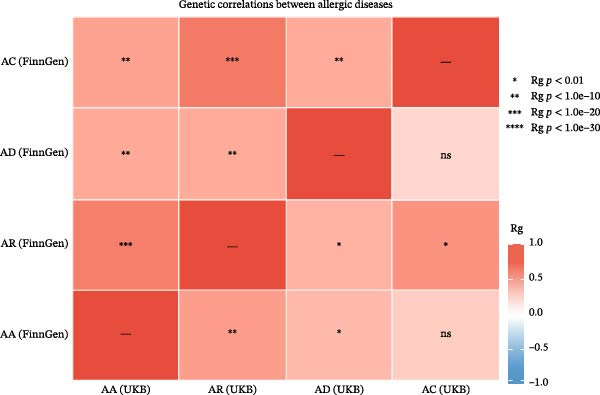
(A)

**Figure:**
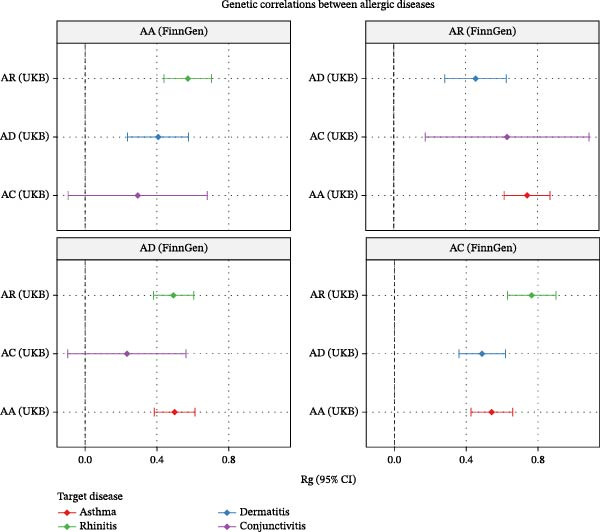
(B)

### 3.2. Exploring the Causal Relationship Among Allergic Diseases

In our LDSC analysis, we identified significant genetic correlations among four pairs of allergic diseases (AA–AD, AA–AR, AR–AD, and AR–AC). Subsequently, each pair was subjected to bidirectional MR‐RAPS analyses and validated in an independent cohort to ensure robustness, ultimately revealing six pairs of allergic diseases with significant causal relationships.

**Figure 3:**
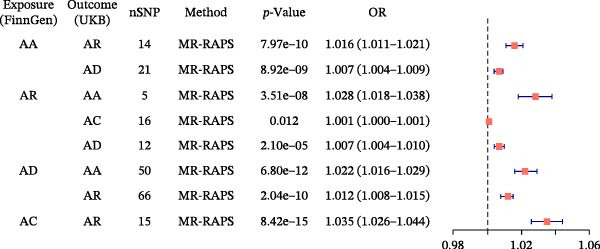
Discovery‐stage MR‐RAPS causal estimates among allergic diseases. Forest plot summarizing two‐sample MR‐RAPS results in the discovery cohort using FinnGen exposures and UK Biobank outcomes.

In the discovery cohort, MR‐RAPS revealed bidirectional causal effects among allergic diseases. Genetically predicted asthma increased the risk of rhinitis (OR 1.016 [1.011–1.021], *p* = 7.97 × 10^−10^) and AD (OR 1.007 [1.004–1.009], *p* = 8.92 × 10^−9^). Conversely, AR raised the risk of AA (OR 1.028 [1.018–1.038], *p* = 3.51 × 10^−8^), AC (OR 1.001 [1.000–1.001], *p* = 0.0117), and AD (OR 1.007 [1.004–1.010], *p* = 2.10 × 10^−5^). Notably, however, the association between AR and AC did not remain significant after Bonferroni correction. AD increased the risks of AA (OR 1.022 [1.016–1.029], *p* = 6.80 × 10^−12^) and AR (OR 1.012 [1.008–1.015], *p* = 2.04 × 10^−10^). Meanwhile, AC elevated AR risk (OR 1.035 [1.026–1.044], *p* = 8.42 × 10^−15^) (Figure 3).

In the validation analysis, all tested allergic disease pairs again showed significant causal effects, with the exception of the AC–AR pair. Based on predefined criteria, six robust disease combinations (AA–AR, AA–AD, AR–AA, AR–AD, AD–AA, and AD–AR) were identified and advanced to the subsequent mediation analysis. Detailed MR‐RAPS results for both analyses are summarized in Supporting Information Table S2 and S3.

### 3.3. MVMR Analysis of Allergic Diseases Adjusted for Type 2 Inflammation

Next, we applied a highly rigorous MVMR model to the six allergic disease pairs that remained significant in both the discovery and validation cohorts. To robustly test for independent causal effects, we comprehensively adjusted for eight type 2 inflammation markers (eosinophil count, IgE, IL‐4, IL‐5, IL‐13, TSLP, IL‐33, and IL‐25) and the presence of the third major comorbid allergic disease. As summarized in Table 2, all six disease pairs continued to confer a significantly increased risk even after this extensive adjustment. This finding strongly suggests that, beyond the widely recognized role of the type 2 inflammatory axis and the potential confounding from co‐occurring allergic conditions, additional biological pathways must drive the progression and interconversion of allergic diseases. Detailed MVMR results are summarized in Supporting Information Table S4.

**Table 2:** Multivariable MR analysis of causal relationships among allergic diseases adjusted for type 2 inflammation markers.

Exposure	Outcome	nSNP	*p*‐Value (IVW)	OR (95% CI)
AA	AD	349	2.62 × 10^−8^	1.007 (1.005–1.009)
AA	AR	349	3.49 × 10^−56^	1.024 (1.021–1.027)
AD	AA	350	7.87 × 10^−27^	1.045 (1.036–1.053)
AD	AR	350	1.25 × 10^−23^	1.020 (1.016–1.024)
AR	AA	353	1.17 × 10^−50^	1.058 (1.050–1.066)
AR	AD	353	3.34 × 10^−13^	1.008 (1.006–1.010)

### 3.4. Immune Cell Trait Alterations by Allergic Diseases and Their Mediating Effects

In the preceding analyses, we identified six allergic disease pairs with robust causal relationships that remained significant after MVMR adjustment for type 2 inflammation markers. To uncover which immune cell traits mediate these relationships, we conducted TSMR analyses using AA, AR, and AD as exposures and 731 distinct immune cell phenotypes as outcomes. Following our rigorous validation and filtering process, we identified several significant immune cell mediators. Significant associations between exposures and immune cell traits are depicted in Figure 4, with complete results provided in Supporting Information Table S5 and S6.

Figure 4Forest plots of causal associations between allergic disease and immune cell trait. (A) The forest plot shows the causal associations of allergic asthma (AA) with immune cell phenotypes. (B) The forest plot shows the causal associations of allergic rhinitis (AR) with immune cell phenotypes. (C) The forest plot shows the causal associations of atopic dermatitis (AD) with immune cell phenotypes.

**Figure:**
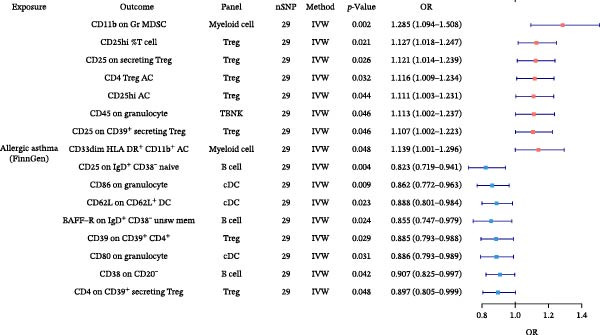
(A)

**Figure:**
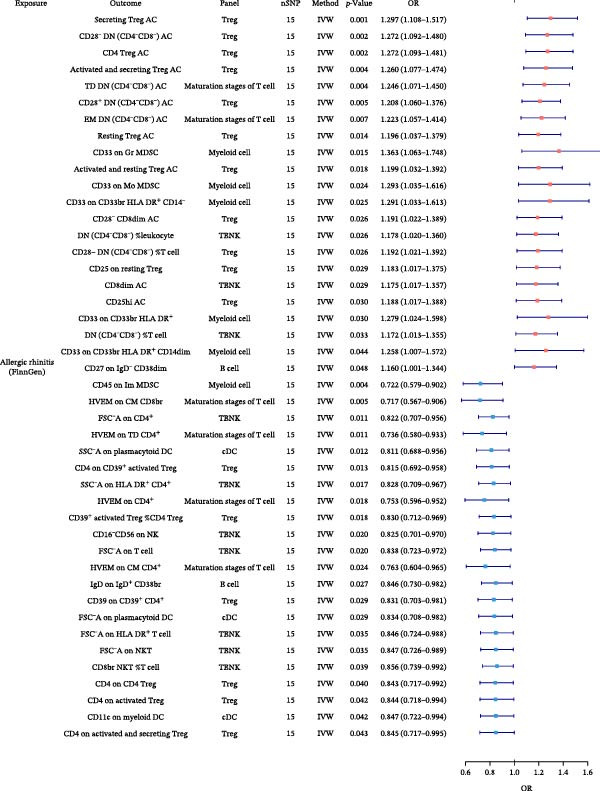
(B)

**Figure:**
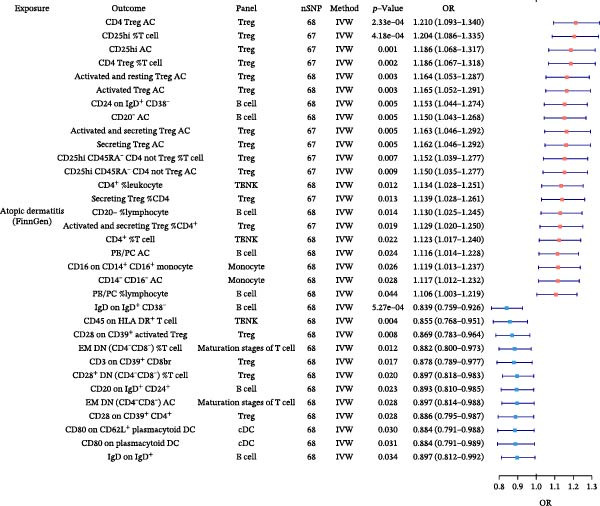
(C)

**Figure 5:**
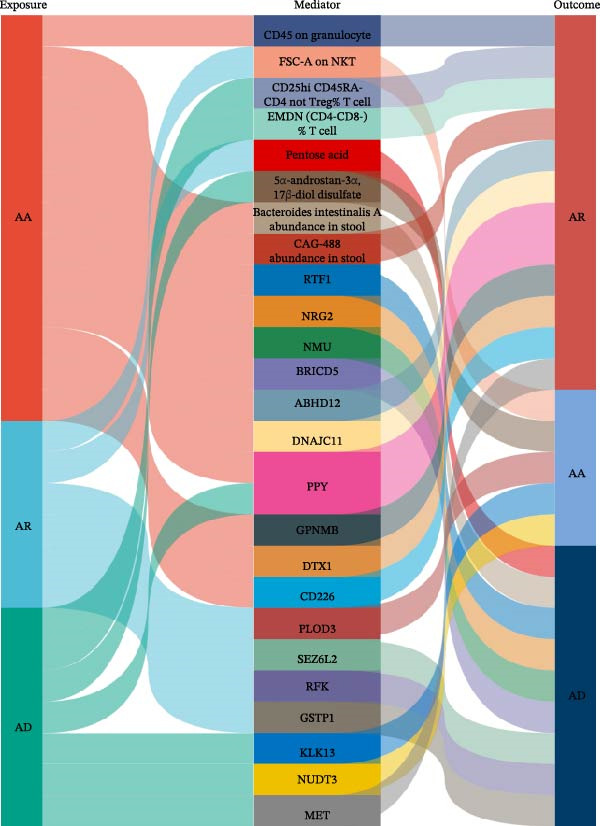
Visualization of the exposure–mediator–outcome relationships using a Sankey diagram.

For each exposure–cell–outcome triad, we then calculated the mediation effect and proportion mediated as detailed in Table 3 and Figure 5. The effect of AA on AR is mediated 1.78% by CD45 expression on granulocytes. Along the AR–AA axis, FSC‐A on NKT cells mediates 3.01%. Additionally, in the AD–AR direction, CD25hi CD45RA– CD4^+^ non‐Treg T cells mediate 2.61% and EM DN T cells mediate 2.20%. Detailed results are provided in Supporting Information Table S7 and S8.

**Table 3:** Mediation effects of immune cell traits on causal links between allergic diseases.

Exposure	Mediator	Outcome	Category	Effect of exposure on outcome β1	Effect of exposure on mediator β2	Effect of mediator on outcome β3	Mediation effect (β2 × β3)	Mediated proportion (%) (β2 × β3)/β1
AA	CD45 on granulocyte	AR	TBNK	0.0160	0.1074	0.0026	0.0003	1.78
AR	CD33 on CD33br HLA DR + CD14dim	AA	Myeloid cell	0.0277	0.2295	0.0012	0.0003	1.03
AR	SSC‐A on HLA DR + CD4+	AA	TBNK	0.0277	−0.1888	−0.0029	0.0006	2.01
AR	FSC‐A on NKT	AA	TBNK	0.0277	−0.1660	−0.0050	0.0008	3.01
AR	CD33 on Gr MDSC	AD	Myeloid cell	0.0069	0.3100	0.0010	0.0003	4.63
AR	CD33 on CD33br HLA DR + CD14‐	AD	Myeloid cell	0.0069	0.2552	0.0006	0.0002	2.37
AR	CD33 on CD33br HLA DR+	AD	Myeloid cell	0.0069	0.2463	0.0006	0.0002	2.18
AR	CD33 on CD33br HLA DR + CD14dim	AD	Myeloid cell	0.0069	0.2295	0.0006	0.0001	1.95
AD	CD25hi CD45RA‐ CD4 not Treg %T cell	AR	Treg	0.0116	0.1416	0.0021	0.0003	2.61
AD	EM DN (CD4‐CD8‐) %T cell	AR	Maturation stages of T cell	0.0116	−0.1251	−0.0020	0.0003	2.20

### 3.5. Plasma Metabolite Alterations by Allergic Diseases and Their Mediating Effects in Causal Networks

Next, we investigated how allergic diseases alter circulating metabolites and which of these molecular changes may serve as mediators in the causal relationships among allergic diseases. Using TSMR, we first identified metabolic traits causally influenced by allergic phenotypes (Figure 6 and Supporting Information Table S9 and S10). Subsequently, we conducted mediation analyses on all causal pathways comprising an initiating allergic phenotype, a metabolite mediator, and a downstream allergic phenotype that satisfied our directional consistency criteria (Table 4 and Figure 5). We identified several plasma metabolites as robust mediators that satisfied our strict validation criteria. The analyses revealed that the effect of AR on AD was mediated 5.60% by pentose acid, whereas the effect of AD on AA was mediated 1.52% by 5α‐androstan‐3α, 17β‐diol disulfate, highlighting specific metabolite intermediates in these networks. Detailed results are provided in Supporting Information Table S11 and S12.

Figure 6Forest plots of causal associations between allergic disease and plasma metabolite. (A) The forest plot shows the causal associations of allergic asthma (AA) with metabolites. (B) The forest plot shows the causal associations of allergic rhinitis (AR) with metabolites. (C) The forest plot shows the causal associations of atopic dermatitis (AD) with metabolites.

**Figure:**
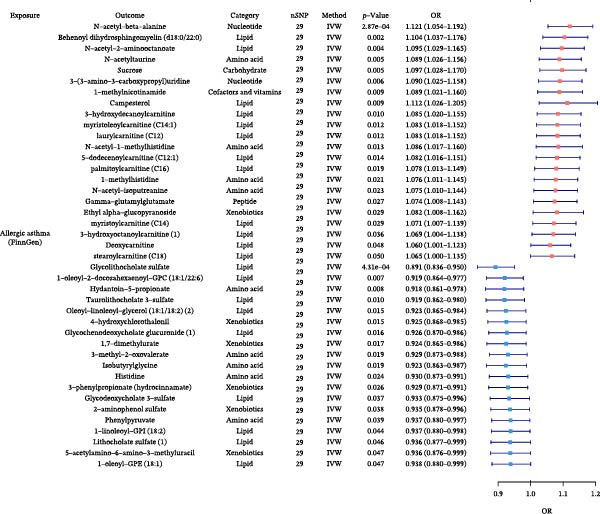
(A)

**Figure:**
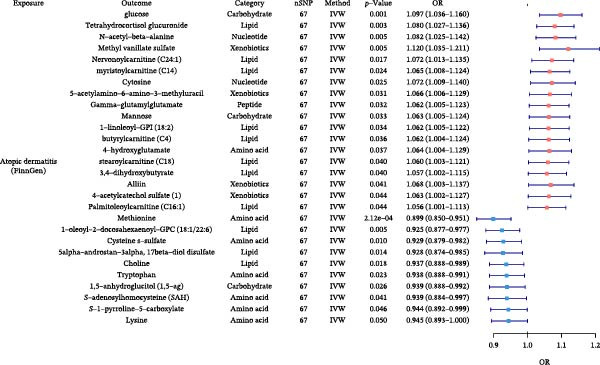
(B)

**Figure:**
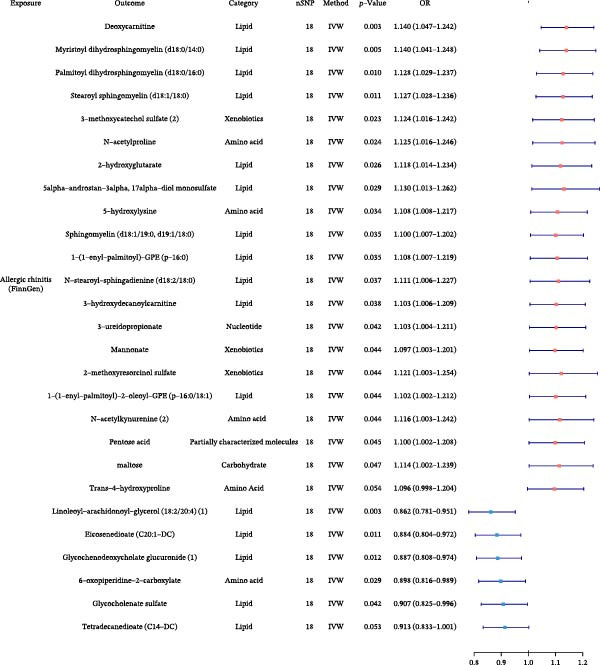
(C)

**Table 4:** Mediation effects of plasma metabolite on causal links between allergic diseases.

Exposure	Mediator	Outcome	Category	Effect of exposure on outcome β1	Effect of exposure on mediator β2	Effect of mediator on outcome β3	Mediation effect (β2 × β3)	Mediated proportion (%) (β2 × β3)/β1
AR	Pentose acid	AD	Partially characterized molecules	0.0069	0.0955	0.0041	0.0004	5.60
AD	5α‐Androstan‐3α, 17β‐diol disulfate	AA	Lipid	0.0222	−0.0750	−0.0045	0.0003	1.52

### 3.6. Gut Microbiota Alterations by Allergic Diseases and Their Mediating Effects in Causal Networks

To further elucidate the downstream effects of allergic diseases, we next examined how these conditions influence the gut microbiota and whether microbiota‐related alterations mediate the causal links among allergic phenotypes. Using TSMR analyses, we identified microbial taxa whose abundances were causally affected by AA, AR, and AD (Figure 7 and Supporting Information Table S13 and S14). Our analysis also revealed specific gut microbiota taxa that acted as validated mediators in the causal networks. Furthermore, we extended our mediation framework to gut microbiota traits, focusing on *Bacteroides intestinalis* A abundance and CAG‐488 abundance in stool as potential intermediates. The effect of AA on AD was mediated 5.21% by *B. intestinalis* A abundance, while the effect of AA on AR was mediated 2.01% by CAG‐488 abundance, underscoring the mediating function of these microbial taxa within the causal relationships among allergic diseases (Table 5 and Figure 5). Detailed results of mediation analysis are provided in Supporting Information Table S15 and S16.

Figure 7Forest plots of causal associations between allergic disease and gut microbiota. (A) The forest plot shows the causal associations of allergic asthma (AA) with gut microbiota. (B) The forest plot shows the causal associations of allergic rhinitis (AR) with gut microbiota. (C) The forest plot shows the causal associations of atopic dermatitis (AD) with gut microbiota.

**Figure:**
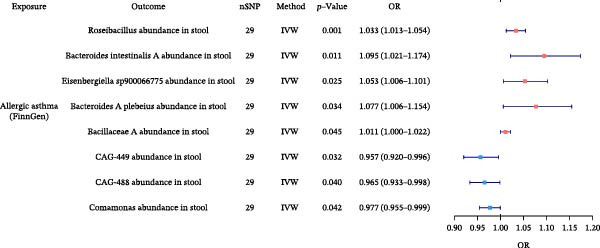
(A)

**Figure:**
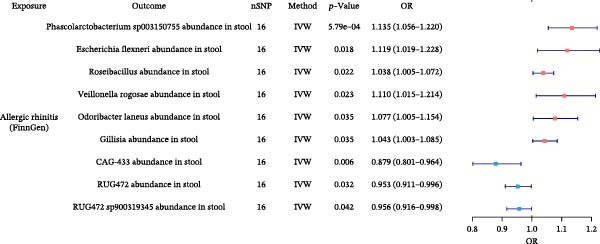
(B)

**Figure:**
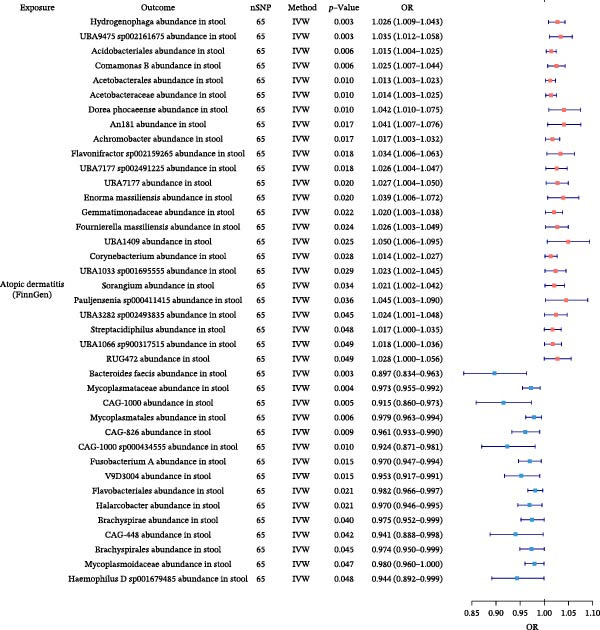
(C)

**Table 5:** Mediation effects of gut microbiota on causal links between allergic diseases.

Exposure	Mediator	Outcome	Effect of exposure on outcome β1	Effect of exposure on mediator β2	Effect of mediator on outcome β3	Mediation effect (β2 × β3)	Mediated proportion (%) (β2 × β3)/β1
AA	*Bacteroides intestinalis* A abundance in stool	AD	0.0066	0.0908	0.0038	0.0003	5.21
AA	CAG‐488 abundance in stool	AR	0.0160	−0.0357	−0.0090	0.0003	2.01

### 3.7. Serum Protein Alterations by Allergic Diseases and Their Mediating Effects in Causal Networks

To comprehensively characterize systemic molecular alterations, we performed TSMR analyses to evaluate the causal impact of allergic diseases on serum protein levels. CALN1 emerged as the most significantly altered protein by AA, exhibiting the highest OR and lowest *p*‐value in the volcano plot (Figure 8A). KEGG analysis highlighted cytokine–cytokine receptor interactions, chemokine signaling, and IL‐17 pathways (Figure 8B). GO enrichment (Figure 8C) prioritized pathways directly relevant to immune cells, including leukocyte migration, eosinophil chemotaxis, lymphocyte migration, chemokine activity, and cytokine activity. In the PPI network, FN1, FYN, SHC1, and CD4 formed central hubs (Figure 8D), and the TF–miRNA regulatory network uncovered potential TFs and miRNAs that jointly regulate key target genes (Figure 8E).

Figure 8TSMR analysis and functional characterization of serum proteins altered by allergic diseases. (A–E) Allergic asthma (AA)–associated proteins: (A) volcano plot of AA effects on serum proteins, (B) KEGG pathway enrichment, (C) GO biological process enrichment, (D) protein–protein interaction (PPI) network (hub proteins in red), and (E) transcription factor–miRNA regulatory network (hub genes in center). (F–J) Allergic rhinitis (AR)–associated proteins: (F) volcano plot of AR effects, (G) KEGG enrichment, (H) GO enrichment, (I) PPI network, and (J) TF–miRNA network. (K–O) Atopic dermatitis (AD)–associated proteins: (K) volcano plot of AD effects, (L) KEGG enrichment, (M) GO enrichment, (N) PPI network, and (O) TF–miRNA network.

**Figure:**
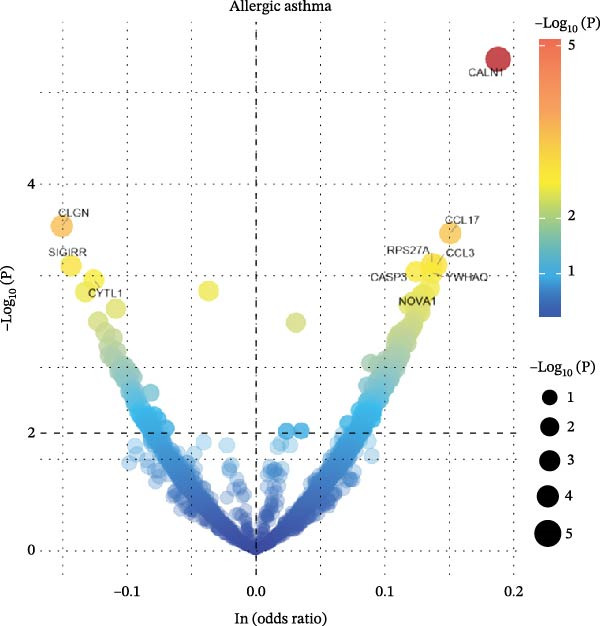
(A)

**Figure:**
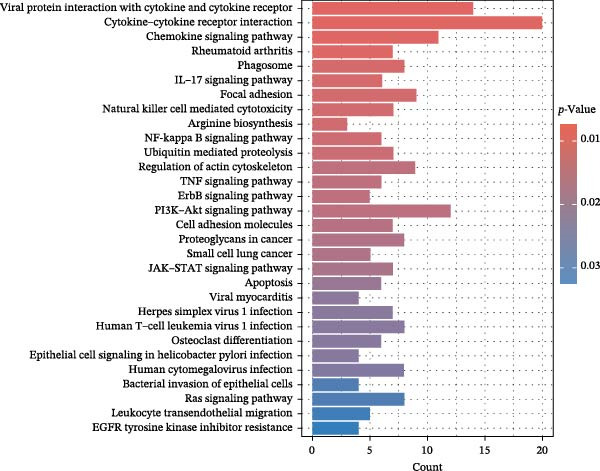
(B)

**Figure:**
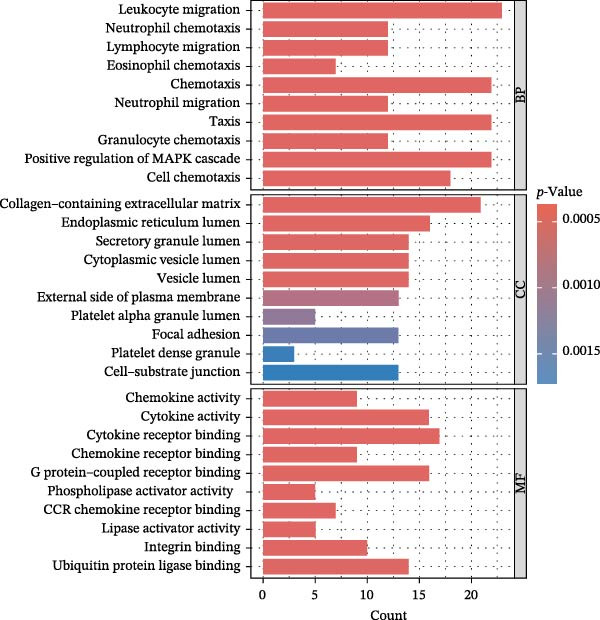
(C)

**Figure:**
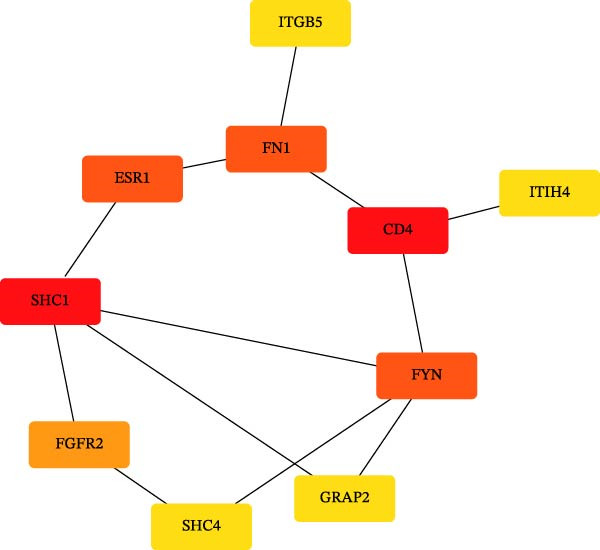
(D)

**Figure:**
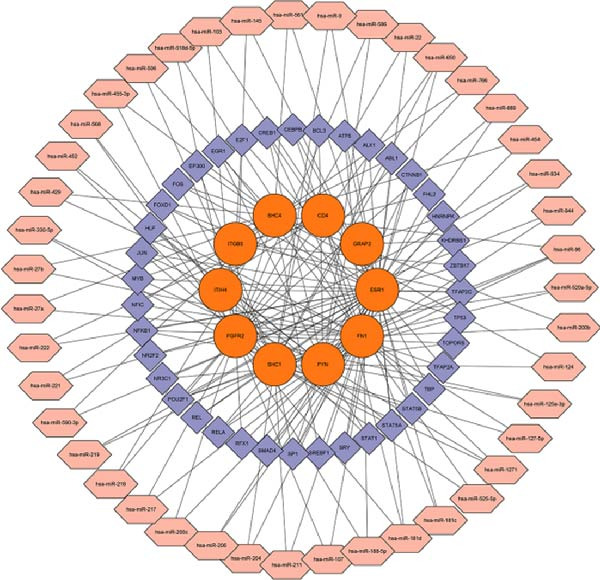
(E)

**Figure:**
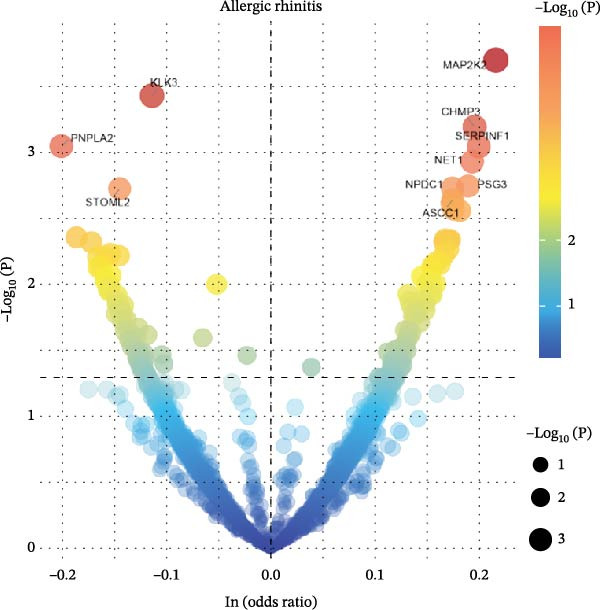
(F)

**Figure:**
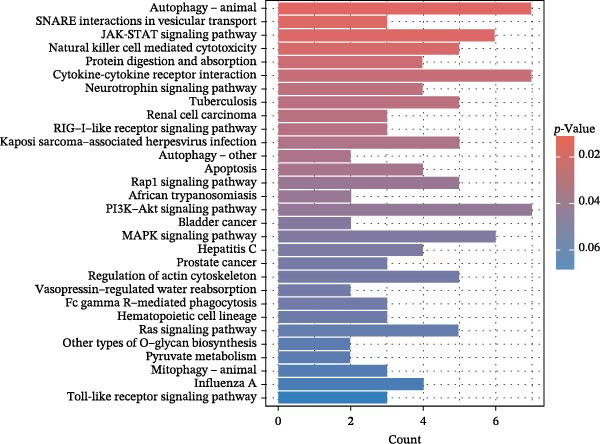
(G)

**Figure:**
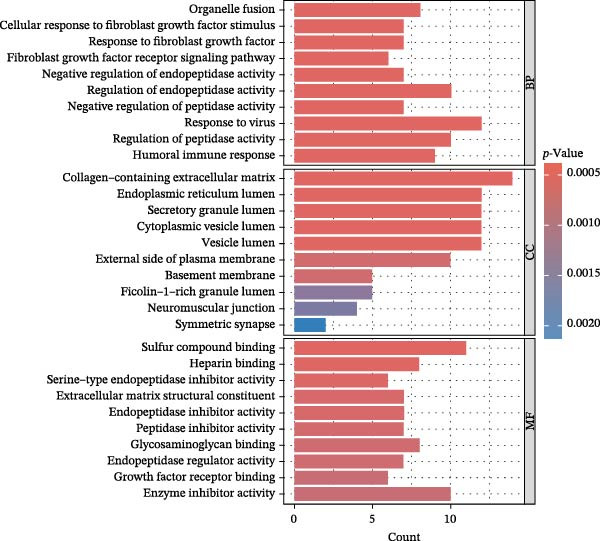
(H)

**Figure:**
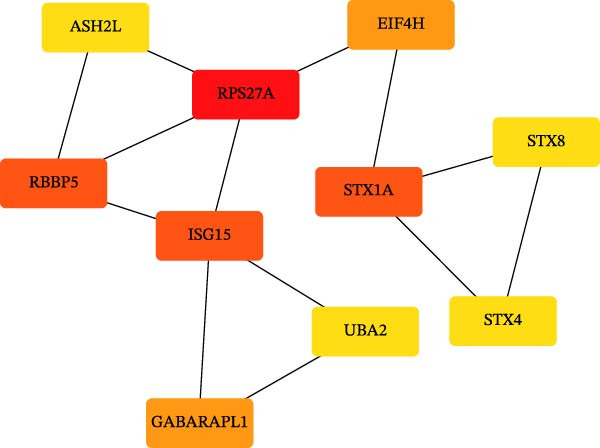
(I)

**Figure:**
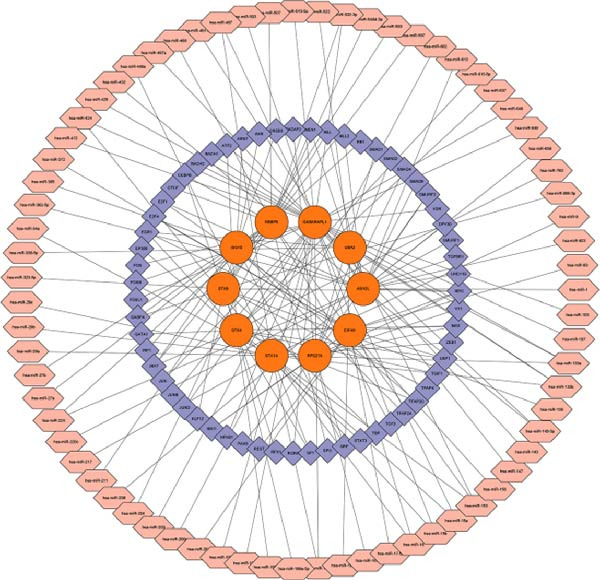
(J)

**Figure:**
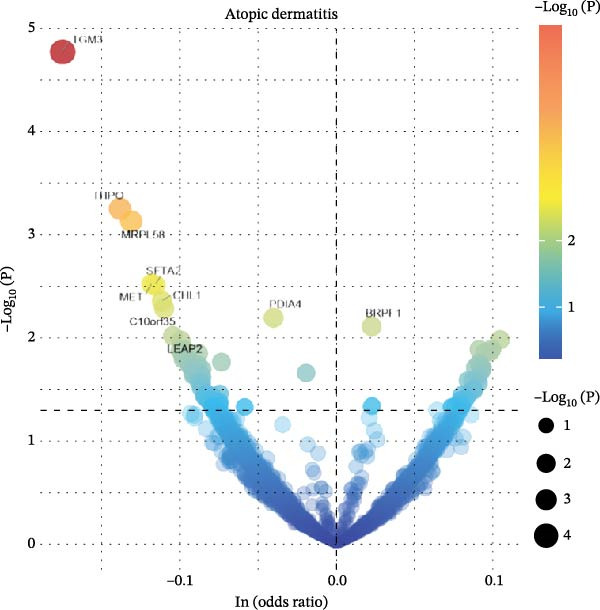
(K)

**Figure:**
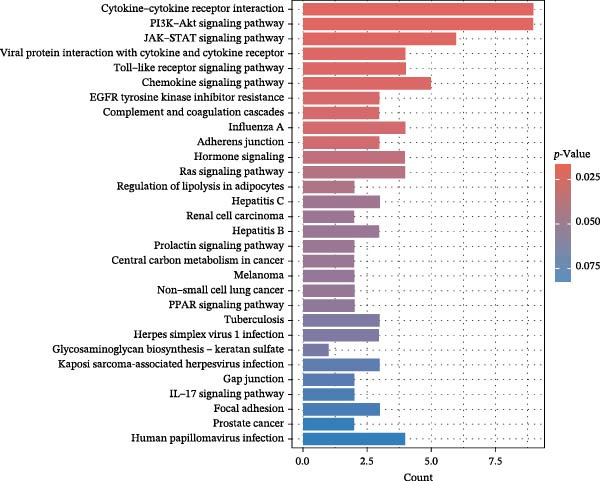
(L)

**Figure:**
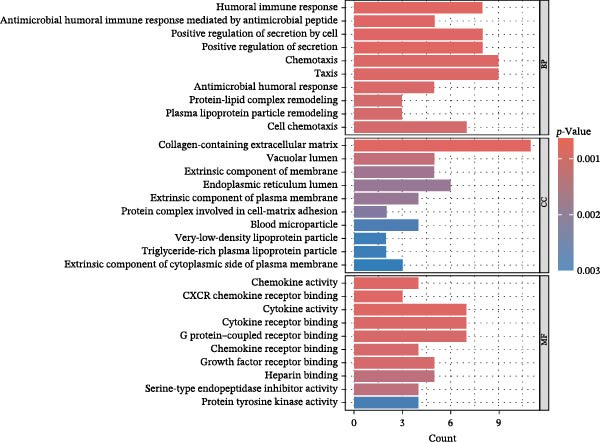
(M)

**Figure:**
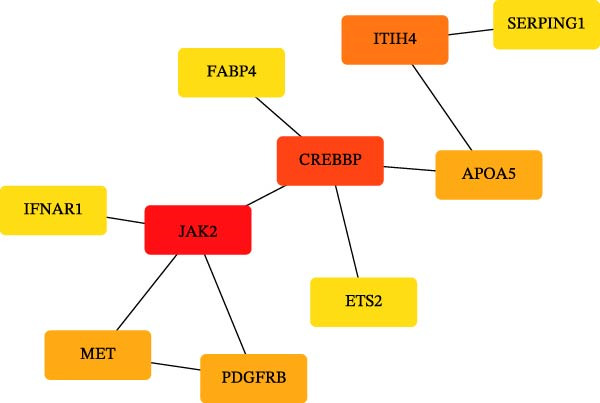
(N)

**Figure:**
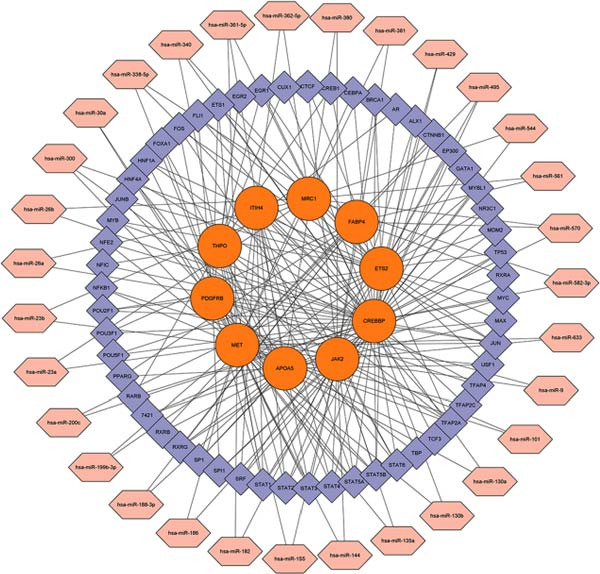
(O)

In AR, MAP2K2 and KLK3 were remarkably altered proteins (Figure 8F). KEGG analysis underscored autophagy–animal, cytokine–cytokine receptor interaction, JAK–STAT signaling, and natural killer cell–mediated cytotoxicity (Figure 8G), while GGO enrichment (Figure 8H) highlighted pathways in fibroblast growth factor signaling, including fibroblast growth factor stimulus, response to fibroblast growth factor, and fibroblast growth factor receptor signaling pathway, as well as humoral immune response. PPI mapping nominated RBBP5, STX1A, and ISG15 as key nodes (Figure 8I), and the TF–miRNA network positioned the key regulators of the hub genes (Figure 8J).

For AD, TGM3 was the foremost perturbed proteins (Figure 8K). KEGG analysis prioritized cytokine–cytokine receptor interaction, PI3K–Akt, and JAK–STAT pathways (Figure 8L), while GO enrichment highlighted humoral immune response, positive regulation of secretion cytokine activity, CXCR chemokine receptor binding, and cell chemotaxis (Figure 8M). In the PPI network (Figure 8N), CREBBBP and JAK2 emerged as hubs. Integration of TF and miRNA interactions revealed candidate regulators of the hub genes (Figure 8O). The details of the results of MR analysis between allergic diseases and serum proteins are shown in Supporting Information Table S17 and S18.

Across the three allergic phenotypes, both shared and disease‐specific alterations in circulating serum proteins were observed. Common features across AA, AR, and AD included the enrichment of cytokine–cytokine receptor interactions and JAK–STAT and PI3K–Akt pathways, signaling axes that collectively reflect a conserved immune‐inflammatory framework underlying allergic disorders. Despite these shared immunoregulatory features, each disease exhibited distinct proteomic signatures reflecting its dominant pathogenic mechanisms. In AA, enrichment was primarily related to immune functions such as leukocyte migration, cytokine activity, and chemokine receptor binding, indicating the pivotal role of coordinated immune‐cell trafficking and intercellular communication in asthma pathogenesis. In AR, the involvement of fibroblast growth factor and autophagy‐related pathways suggested epithelial remodeling and mucosal immune adaptation. In contrast, AD showed distinct enrichment in adhesion and junction‐related pathways, including adherens junction, focal adhesion, and gap junction, suggesting enhanced epithelial barrier signaling. Together, these findings delineate a shared immune‐cytokine signaling core that links allergic diseases, while the disease‐specific proteomic patterns highlight the diverse downstream biological processes driving organ‐specific manifestations and cross‐disease heterogeneity.

Next, we evaluated the mediating roles of circulating serum proteins in the causal relationships among allergic diseases. In the causal pathway from AA to AD, NRG2 mediated 3.91% of the total effect, followed by NMU (3.71%), ABHD12 (2.76%), and RTF1 (2.27%). Along the pathway from AA to AR, ABHD12 accounted for the largest indirect contribution at 3.31% of the total effect, followed by ITGB5 (2.05%), CD226 (1.78%), DNAJC11 (1.76%), GPNMB (1.66%), and DTX1 (1.37%). In the AR to AA direction, PLOD3 mediated 1.44% of the causal estimate. For the causal pathway from AR to AD, SEZ6L2 mediated the largest proportion at 6.57% of the total effect, followed by RFK at 4.58% and GSTP1 at 3.63%. For the causal pathway from AD to AA, NUDT3 mediated 1.78% of the total effect, and KLK13 mediated 0.87%. Within the causal link between AD and AR, MET and PPY each accounted for 1.78% mediation (Table 6 and Figure 5). Detailed results of mediation analysis are provided in Supporting Information Table S19 and S20.

**Table 6:** Mediation effects of serum proteins on causal links between allergic diseases.

Exposure	Mediator	Outcome	Effect of exposure on outcome β1	Effect of exposure on mediator β2	Effect of mediator on outcome β3	Mediation effect (β2 × β3)	Mediated proportion (%) (β2 × β3)/β1
AA	RTF1	AD	0.0066	0.0240	0.0062	0.0002	2.27
AA	NRG2	AD	0.0066	0.0819	0.0032	0.0003	3.91
AA	NMU	AD	0.0066	0.0878	0.0028	0.0002	3.71
AA	ITGB5	AD	0.0066	0.1129	0.0030	0.0003	5.15
AA	BRICD5	AD	0.0066	0.0853	0.0021	0.0002	2.76
AA	ABHD12	AR	0.0160	0.0978	0.0054	0.0005	3.31
AA	DNAJC11	AR	0.0160	0.0944	0.0030	0.0003	1.76
AA	PPY	AR	0.0160	−0.0937	−0.0028	0.0003	1.66
AA	MST1	AR	0.0160	0.1091	0.0011	0.0001	0.73
AA	GPNMB	AR	0.0160	−0.1085	−0.0030	0.0003	2.05
AA	DTX1	AR	0.0160	0.0888	0.0025	0.0002	1.37
AA	POSTN	AR	0.0160	0.1099	0.0026	0.0003	1.78
AA	CD226	AR	0.0160	0.0812	0.0040	0.0003	2.05
AR	PLOD3	AA	0.0277	0.1239	0.0032	0.0004	1.44
AR	SEZ6L2	AD	0.0069	−0.1365	−0.0033	0.0005	6.57
AR	RFK	AD	0.0069	0.1161	0.0027	0.0003	4.58
AR	GSTP1	AD	0.0069	0.1153	0.0022	0.0003	3.63
AD	YWHAE	AA	0.0222	−0.0839	−0.0029	0.0002	1.11
AD	KLK13	AA	0.0222	−0.0817	−0.0024	0.0002	0.87
AD	NUDT3	AA	0.0222	0.0785	0.0050	0.0004	1.78
AD	MET	AR	0.0116	−0.0937	−0.0029	0.0003	1.78
AD	PPY	AR	0.0116	−0.0826	−0.0028	0.0002	1.78

## 4. Discussion

In this study, we investigated the genetic correlations and causal relationships among allergic diseases in large‐scale FinnGen and UK Biobank cohorts to map the causal network linking AA, AR, AD, and AC. LDSC established pervasive positive genetic correlations across most disease pairs, corroborating prior epidemiological observations of co‐occurrence. Building on these findings, we are the first to use MR‐RAPS analyses to provide the robust evidence of a causal relationship among allergic diseases in both discovery and validation cohorts. After adjusting for type 2 inflammation–related factors in MVMR, the causal relationships among allergic diseases remained significant, indicating that drivers beyond the canonical Th2 inflammatory axis are at play. We then systematically characterized the impact of allergic diseases on the internal milieu, profiling alterations in immune cells, plasma metabolites, gut microbiota, and serum proteins. Finally, we conducted mediation analyses to investigate the mediator effect of these multiomic alterations in the causal effects between allergic diseases. This represents the first comprehensive MR study to elucidate not only the bidirectional causal relationships among allergic diseases but also the downstream alterations in immune cell composition, plasma metabolome, gut microbiota, and serum proteome and to quantify the mediating roles of these multiomic changes in interdisease causality.

Numerous observational studies have documented strong correlations and high comorbidity among AA, AD, AR, and AC [54–56], but the inherent limitations of clinical observational research prevent the determination of causal directionality. MR analysis overcomes this limitation by leveraging genetic instruments. Ahn et al. first demonstrated bidirectional risk promotion between AD and AA using MR [57], and Wang et al. [58] reported a significant bidirectional association of AD, AC, and AR with AA. However, prior investigations have largely focused on individual phenotype pairs, without building a comprehensive and robust causal network across multiple allergic diseases, without applying multivariable adjustment for key type 2 inflammation–related factors. An international analysis of 12 European birth cohorts further demonstrated that by age eight, half of children with multimorbidity of eczema, rhinitis, and asthma lacked, and specific IgE sensitization to common allergens explained only 38% of these cases, pointing to non‐IgE–mediated mechanisms in the concurrent development of multiple allergic diseases [59]. Therefore, it is crucial to elucidate causal relationships that operate independently of type 2 inflammation. In this study, we applied multivariable adjustment for key Th2 markers and systematically examined how allergic diseases perturb the internal milieu, including immune cells, plasma metabolites, gut microbiota, and serum proteins, and how these multiomic alterations mediate interdisease causal effects.

Many allergy risk genes concurrently regulate multiple immune and inflammatory pathways, acting independently across different tissues or cell types, and thereby cause a single genetic variant to influence diverse allergic phenotypes. Also, our prior work confirmed horizontal pleiotropy among these traits [43]. Previous studies have nonetheless underscored the importance of shared genetic factors in allergic multimorbidity; for example, Gong et al. [60] applied genomic structural equation modeling (Genomic‐SEM) to reveal a common latent factor driving substantial genetic correlation among asthma, eczema, and AR. To address horizontal pleiotropy, we adopted the MR‐RAPS framework, which accounts for pleiotropic effects and allows the inclusion of weaker genetic instruments by weighting them according to the precision of their exposure and outcome associations. Through dual validation in both the discovery and replication cohorts, we ultimately identified six pairs of allergic diseases with causal relationships.

Importantly, our highly adjusted MVMR demonstrated that these cross‐disease effects persist even after accounting for a comprehensive set of eight type 2 inflammation markers and the confounding effect of the third major comorbid allergic disease. This demonstrates a level of causal independence that goes beyond the dominant Th2‐driven axis. However, we acknowledge that even this extensive set of covariates cannot capture all potential confounders, and a degree of residual confounding may remain. Nevertheless, our findings provide strong evidence for the existence of parallel or alternative mechanisms driving these causal relationships. Unsurprisingly, our downstream analyses identified a diverse constellation of molecular and cellular intermediates—including Treg cell subsets, plasma metabolites such as pentose acid and 5α‐androstan‐3α, 17β‐diol disulfate, gut microbial taxa like *B. intestinalis* A and CAG‐488, and numerous circulating proteins.

Allergic diseases may induce alterations in immune cell phenotypes, thereby reshaping both cellular and humoral environments and mediating the onset and progression of secondary allergic conditions. Treg cells play a critical role in maintaining tolerance to allergens at environmental interfaces in the airways, skin, and gut [61]. However, patients with allergic diseases often exhibit impaired CD4^+^CD25^+^Treg–mediated suppression of T helper cell responses to allergens, leading to uncontrolled Th2 inflammation [62]. The gut–lung axis has garnered increasing attention in allergy research. In a neonatal cohort, Stokholm et al. [63] showed that higher abundance of *B. intestinalis* at 1 year of age was associated with elevated asthma risk by age five, implicating early‐life gut dysbiosis in allergic predisposition. Consistent with this, our MR analysis indicates that asthma causally increases *B. intestinalis* levels, which in turn mediates a higher risk of AD. Future work should elucidate the mechanistic pathways by which *B. intestinalis* modulates immune responses and drives interdisease progression.

Beyond microbial influences, metabolic alterations may also bridge distinct allergic phenotypes. According to a randomized, placebo‐controlled metabolomic study in asthma [64], plasma 5α‐androstan‐3α, 17β‐diol disulfate showed no significant change following fluticasone furoate/vilanterol treatment, suggesting it is not directly responsive to corticosteroid intervention. However, its potential involvement in the transition from AD to asthma cannot be excluded and warrants further investigation. Although no specific studies have directly linked pentose acid to allergic diseases, this metabolite may reflect host metabolic reprogramming through the pentose phosphate pathway (PPP), which supports oxidative stress regulation and activation of immune cells, such as neutrophils and macrophages, under allergic conditions [65, 66].

Our mediation analysis revealed that circulating proteins play a crucial bridging role in the interplay between atopic diseases. For instance, ABHD12, a serine hydrolase involved in lipid metabolism, mediated 3.31% of the effect in the causal pathway from AA to AR. ABHD12, a serine hydrolase, plays a critical role in lipid metabolism by hydrolyzing lysophosphatidylserine and the endocannabinoid 2‐arachidonoylglycerol (2‐AG), a major arachidonic acid–derived signaling lipid [67, 68]. ABHD12 may modulate the Th2 immune environment by affecting eicosanoid balance, thus providing a plausible mechanistic link between asthma and AR via lipid‐mediated immune signaling. In the causal pathway from AR to AD, SEZ6L2 exhibited the most significant mediation effect, accounting for 6.57% of the total effect, followed by RFK and GSTP1. SEZ6L2, a member of the SEZ6 protein family, is a type I membrane protein expressed on the cell surface [69]. Although there are currently no studies linking SEZ6L2 to allergic diseases, SEZ6L2 has been implicated in caspase‐dependent apoptosis and the transport of cathepsin D to endosomes, warranting further investigation [69, 70]. RFK and GSTP1 are involved in cellular energy metabolism and oxidative stress response, respectively—mechanisms that are crucial for skin barrier function and chronic inflammation [71, 72].

Based on our two‐step MR analyses, we identified multiple mediators, including immune cell subsets, plasma metabolites, gut microbiota taxa, and plama proteins, that partially explained the causal relationships among allergic diseases. Although each mediator was evaluated independently, accumulating evidence suggests that these layers are biologically interconnected. For example, immune cell dysregulation may serve as the upstream driver that triggers systemic metabolic remodeling and reshapes the gut microbial ecosystem [73]. In addition, gut microbial composition (e.g., abundance of *B. intestinalis*) may secondarily affect host metabolic pathways and circulating metabolites, which in turn modulate immune cell differentiation and activation [74, 75]. Likewise, immune‐metabolic coupling, as described by Rodriguez‐Coira et al. [17], links metabolic reprogramming to immune effector functions. Circulating proteins such as ABHD12 and SEZ6L2, identified in our mediation analysis, may act as downstream effectors integrating immune, metabolic, and microbial perturbations, although direct mechanistic evidence linking them to the microbiota, metabolite, and immune axis remains to be established. Therefore, while our MR framework demonstrates independent causal mediators, these findings collectively support a systemic network in which immune, metabolic, microbial, and proteomic alterations reinforce one another to promote allergic multimorbidity.

Overall, we identified multiple mediators that play a role in the causal relationships among allergic diseases, although the proportion of the total effect explained by each individual mediator was relatively small (ranging from 1% to 6%). Rather than diminishing the significance of these findings, this phenomenon likely reflects the inherent systemic and network characteristics of allergic diseases, where no single pathway exerts a dominant effect. In this context, the identified mediators likely represent key nodes within a robust pathogenic network, acting as the “tip of the iceberg” of broader systemic alterations. Consequently, the transition and progression of these conditions are presumably driven by the cumulative impact of multiple small‐effect pathways involving immune regulation, metabolic alterations, gut microbiota composition, and plasma protein signaling [76, 77]. Several factors account for this: first, the biological redundancy and heterogeneity of allergic diseases make it unlikely that any single mediator bears the majority of the causal load; second, mediators likely exert distributed influences through parallel or convergent pathways. Finally, the progression and transformation of allergic diseases is a dynamic process where mediators may exert phase‐specific effects—for instance, mast cells release mediators in a selective, time‐dependent fashion shaped by underlying signaling cascades [23, 78]. This characteristic also has important implications for clinical translation. Since the causal effects are distributed across multiple pathways, therapeutic interventions targeting a single mediator may yield limited clinical efficacy. Therefore, future strategies for precision medicine should consider multitarget or network‐based approaches to effectively halt the progression and mutual transformation of allergic multimorbidity.

By utilizing large GWAS cohorts and robust IVs, our analysis improved the statistical power for detecting causal relationships and strengthened the reliability of the findings. Nonetheless, important limitations must be acknowledged. The available genetic instruments and overall sample size may not fully capture the polygenic complexity of these conditions, and the chosen variants might incompletely reflect their heritable architectures. Furthermore, while we employed MR‐RAPS specifically to mitigate the impact of horizontal pleiotropy, we cannot definitively exclude the possibility of residual confounding from complex or unmeasured pleiotropic pathways. Another significant limitation involves the potential for residual confounding from factors beyond the adjusted type 2 inflammatory markers. Although our analysis successfully accounted for major confounders such as smoking and BMI, some environmental exposures and medication histories could not be fully addressed due to the nature of summary‐level GWAS data. These unmeasured factors might influence the observed causal estimates. Therefore, our findings should be interpreted with appropriate caution and warrant further validation through experimental studies and large‐scale clinical cohorts. Additionally, we acknowledge that the mediation analysis was exploratory and conducted in a single discovery cohort, with exposures derived from the FinnGen database and outcomes from the UK Biobank, without validation in an independent replication cohort. While the causal links themselves were robustly and reciprocally replicated, the specific mediation pathways identified here represent hypotheses that await validation in independent, swapped‐cohort analyses. Another significant limitation is that our research data were derived exclusively from individuals of European ancestry. This restriction may limit the generalizability of the identified causal networks and mediating pathways to other ethnic groups because genetic architectures and environmental exposures vary across populations. Although some non‐European cohorts provide GWAS summary statistics for allergic diseases, they currently lack the integrated multiomic profiles and the specific type 2 inflammatory markers necessary for our comprehensive mediation and MVMR frameworks. Therefore, caution is warranted when extrapolating these findings to broader populations. Future research should prioritize cross‐ancestry validation as more high‐dimensional, multiomic datasets from diverse racial and ethnic groups become accessible.

Building on these integrated findings, our study provides a genetic foundation for future translational research. The identified mediators may serve as potential biomarkers for predicting allergic disease progression, for example, the transition from AD to asthma, thereby aiding early risk assessment and personalized management. In addition, several mediators identified in this study hold promise as therapeutic targets. The protective microbial taxa CAG‐488 may guide the development of microbiota‐based therapies, such as engineered probiotics or microbial consortia designed to restore immune‐metabolic homeostasis. Similarly, molecules such as NUDT3 could be explored as druggable targets, where modulation through RNA interference, small‐molecule inhibitors, or nucleic acid delivery approaches might prevent or reverse allergic disease conversion. While these applications remain theoretical and require extensive experimental validation, our findings provide a conceptual framework and potential roadmap for future mechanistic and translational investigations into allergic disease progression.

Moreover, several mediators, such as CAG‐488, ITGB5, and NUDT3, could represent therapeutic targets for future intervention. For instance, protective taxa like *B. intestinalis* might inspire probiotic‐based therapies, while targeting molecules such as ITGB5 or NUDT3 via nucleic acid delivery could modulate allergic conversion. Although further experimental validation is required, these findings offer valuable directions for developing mechanism‐based treatment strategies.

In this study, we systematically investigated the genetic correlations and causal relationships among allergic diseases using large‐scale cohorts from FinnGen and the UK Biobank, aiming to construct a comprehensive causal network linking AA, AR, AD, and AC. To our knowledge, this represents one of the most extensive explorations of causal interplay among allergic diseases to date. Furthermore, by incorporating MVMR to adjust for type 2 inflammation–related factors, we evaluated the independent effects of allergic diseases on internal homeostasis. Importantly, we focused on identifying mediating factors beyond type 2 inflammation, performing a broad and integrative analysis of potential mediators, their biological pathways, regulatory mechanisms, and downstream targets. These findings offer novel insights into the shared and distinct molecular underpinnings of allergic diseases and provide a valuable resource for identifying therapeutic targets and biomarkers.

## Author Contributions

**Ping-An Zhang**: conceptualization, writing – original draft, investigation. **Jie-Lin Wang**: software, data curation, writing – original draft. **Shi-Yan Fu**: data curation. **Hua-Lian Luo**: formal analysis. **Nai-Jian Li**: formal analysis. **Run-Dong Qin**: writing – review and editing, formal analysis. **Jing Li**: supervision, writing – review and editing, funding acquisition.

## Funding

This work was supported by the Noncommunicable Chronic Diseases‐National Science and Technology Major Project (Grant 2024ZD0529900), the National Natural Science Foundation of China (Grant 82161138020), the Major Project of Guangzhou National Laboratory (Grant GZNL2024A02002), and the Guangdong Innovation Team Project of General College and University (Grant 2023KCXTD024).

## Disclosure

All authors read and approved the final manuscript.

## Ethics Statement

The data for this investigation were acquired from previously published studies and public sources, negating the need for further ethical approval.

## Conflicts of Interest

The authors declare no conflicts of interest.

## Supporting Information

Additional supporting information can be found online in the Supporting Information section.

## Supporting information


**Supporting Information 1** The completed STROBE‐MR checklist for this study, providing detailed information on the reporting of our Mendelian randomization analysis.

**Supporting Information 2** Table S1: Genetic correlations among allergic diseases. Table S2: The IVs used in Mendelian randomization analysis among allergic diseases. Table S3: The results of MR‐RAPS analysis among allergic diseases. Table S4: The results of MVMR analysis among allergic diseases. Table S5: The IVs used in Mendelian randomization analysis of allergic diseases on immune cell traits. Table S6: The results of Mendelian randomization analysis of allergic diseases on immune cell traits. Table S7: The IVs used in Mendelian randomization analysis of the causal effects of immune cell traits on allergic diseases. Table S8: Detailed results of the Mendelian randomization analysis assessing the causal effects of immune cell traits on allergic diseases. Table S9: The IVs used in Mendelian randomization analysis of allergic diseases on plasma metabolites. Table S10: The results of Mendelian randomization analysis of allergic diseases on plasma metabolites. Table S11: The IVs used in Mendelian randomization analysis of the causal effects of plasma metabolites on allergic diseases. Table S12: Detailed results of the Mendelian randomization analysis assessing the causal effects of plasma metabolites on allergic diseases. Table S13: The IVs used in Mendelian randomization analysis of allergic diseases on gut microbiota. Table S14: The results of Mendelian randomization analysis of allergic diseases on gut microbiota. Table S15: The IVs used in Mendelian randomization analysis of the causal effects of gut microbiota on allergic diseases. Table S16: Detailed results of the Mendelian randomization analysis assessing the causal effects of gut microbiota on allergic diseases. Table S17: The IVs used in Mendelian randomization analysis of allergic diseases on serum proteins. Table S18: The results of Mendelian randomization analysis of allergic diseases on serum proteins. Table S19: The IVs used in Mendelian randomization analysis of the causal effects of serum proteins on allergic diseases. Table S20: Detailed results of the Mendelian randomization analysis assessing the causal effects of serum proteins on allergic diseases. Table S21: Detailed results of the causal effect of potential mediators on allergic diseases.

## Data Availability

The data that support the findings of this study are available in the FinnGen repository (https://www.finngen.fi/en) and IEU Open GWAS project (https://www.ebi.ac.uk/gwas/downloads/summary-statistics) and GWAS Catalog (https://www.ebi.ac.uk/gwas/downloads/summary-statistics).
