# Supplementary material for: Genetic Correlations and Causal Relationships Among Allergic Diseases: A Comprehensive Mendelian Randomization Study With Multiomic Mediation Analysis
Source: Mediators Inflamm. 2026 Apr 9;2026:5466012. doi: 10.1155/mi/5466012 (PMC13063389; doi:10.1155/mi/5466012)
Supplement: Supplementary file 1 — Supporting Information 1 The completed STROBE‐MR checklist for this study, providing detailed information on the reporting of our Mendelian randomization analysis. [file MI-2026-5466012-s001.docx]

**STROBE-MR checklist of recommended items to address in reports of Mendelian randomization studies**^1^ ^2^

| **Item No.** | **Section** | **Checklist item** | **Page No.** | **Relevant text from manuscript** |
| --- | --- | --- | --- | --- |
| 1 | **TITLE and ABSTRACT** | Indicate Mendelian randomization (MR) as the study’s design in the title and/or the abstract if that is a main purpose of the study | 1-2 | "Mendelian randomization" is named in the title and abstract |
|  | **INTRODUCTION** |  |  |  |
| 2 | **Background** | Explain the scientific background and rationale for the reported study. What is the exposure? Is a potential causal relationship between exposure and outcome plausible? Justify why MR is a helpful method to address the study question | 4-6 | In the “Introduction” section, we discussed why we anlazyes the causal relationships among allergic diseases. In the fourth paragraph of the Introduction section, we discuss why Mendelian randomization is useful. |
| 3 | **Objectives** | State specific objectives clearly, including pre-specified causal hypotheses (if any). State that MR is a method that, under specific assumptions, intends to estimate causal effects | 4-6 | In the forth paragraph of “Introduction”, we describe the causal hypotheses of our study |
|  | **METHODS** |  |  |  |
| 4 | **Study design and data sources** | Present key elements of the study design early in the article. Consider including a table listing sources of data for all phases of the study. For each data source contributing to the analysis, describe the following: | 7 | The "Data Sources" section of the Research Design and Methods section provides existing information about GWAS research. For more information, please refer to GWAS's original publication. |
|  | a) | Setting: Describe the study design and the underlying population, if possible. Describe the setting, locations, and relevant dates, including periods of recruitment, exposure, follow-up, and data collection, when available. | 7 |  |
|  | b) | Participants: Give the eligibility criteria, and the sources and methods of selection of participants. Report the sample size, and whether any power or sample size calculations were carried out prior to the main analysis | 7 |  |
|  | c) | Describe measurement, quality control and selection of genetic variants | 7 |  |
|  | d) | For each exposure, outcome, and other relevant variables, describe methods of assessment and diagnostic criteria for diseases | 7 |  |
|  | e) | Provide details of ethics committee approval and participant informed consent, if relevant | 7 |  |
| 5 | **Assumptions** | Explicitly state the three core IV assumptions for the main analysis (relevance, independence and exclusion restriction) as well assumptions for any additional or sensitivity analysis | 6-10 | The three core IV assumptions explained in the "Study design" section.The main analysis and sensitivity analysis are explained in the section 2.3 and 2.4. |
| 6 | **Statistical methods: main analysis** | Describe statistical methods and statistics used | 6-10 |  |
|  | a) | Describe how quantitative variables were handled in the analyses (i.e., scale, units, model) | 7 | a)We refer to the relevant data description of GWAs public databases. |
|  | b) | Describe how genetic variants were handled in the analyses and, if applicable, how their weights were selected | 8 | b) We will describe it in the "2.3 Selection of instrumental variables" section. |
|  | c) | Describe the MR estimator (e.g. two-stage least squares, Wald ratio) and related statistics. Detail the included covariates and, in case of two-sample MR, whether the same covariate set was used for adjustment in the two samples |  | We did not include covariates. |
|  | d) | Explain how missing data were addressed | 7-8 | d) We described the screening criteria for SNPs and explained the use of independent SNPs in the "2.3 Selection of instrumental variables" section. |
|  | e) | If applicable, indicate how multiple testing was addressed |  | e) N/A |
| 7 | **Assessment of assumptions** | Describe any methods or prior knowledge used to assess the assumptions or justify their validity | 8-10 | We described the testing methods for tool heterogeneity and level ambiguity in the "2.4. Statistical analysis" section |
| 8 | **Sensitivity analyses and additional analyses** | Describe any sensitivity analyses or additional analyses performed (e.g. comparison of effect estimates from different approaches, independent replication, bias analytic techniques, validation of instruments, simulations) | 8-10 | We have described the relevant content in the "2.4. Statistical analysis" section. |
| 9 | **Software and pre-registration** |  |  |  |
|  | a) | Name statistical software and package(s), including version and settings used | 10 | We carried out the MR analysis using the R version 4.3.1 software's "TwoSampleMR" package(version 0.5.7. ) and “MendelianRandomization” (version 0.10.0). |
|  | b) | State whether the study protocol and details were pre-registered (as well as when and where) |  | The study protocol and details was not pre-registered. |
|  | **RESULTS** |  |  |  |
| 10 | **Descriptive data** |  |  |  |
|  | a) | Report the numbers of individuals at each stage of included studies and reasons for exclusion. Consider use of a flow diagram | 7 | The relevant information is listed in the "Data Sources" section |
|  | b) | Report summary statistics for phenotypic exposure(s), outcome(s), and other relevant variables (e.g. means, SDs, proportions) | 10-14 | Relevant information is presented in Supplemental tables |
|  | c) | If the data sources include meta-analyses of previous studies, provide the assessments of heterogeneity across these studies |  | N/A |
|  | d) | For two-sample MR:  i.  Provide justification of the similarity of the genetic variant-exposure associations between the exposure and outcome samples  ii.  Provide information on the number of individuals who overlap between the exposure and outcome studies | 7 | Our samples do not overlap and are explained in the "Data Sources" section |
| 11 | **Main results** |  |  |  |
|  | a) | Report the associations between genetic variant and exposure, and between genetic variant and outcome, preferably on an interpretable scale | 10-14 | In the "Results" section, we presented the relevant results through graphs and tables, with additional text explanations. |
|  | b) | Report MR estimates of the relationship between exposure and outcome and the measures of uncertainty from the MR analysis on an interpretable scale, such as odds ratio or relative risk per SD difference | 10-14 |  |
|  | c) | If relevant, consider translating estimates of relative risk into absolute risk for a meaningful time period | 10-14 |  |
|  | d) | Consider plots to visualize results (e.g. forest plot, scatterplot of associations between genetic variants and outcome versus between genetic variants and exposure) | 10-14 |  |
| 12 | **Assessment of assumptions** |  |  |  |
|  | a) | Report the assessment of the validity of the assumptions | 8-10 | The method of sensitivity analysis is explained in the "2.4. Statistical analysis" section. |
|  | b) | Report any additional statistics (e.g., assessments of heterogeneity across genetic variants, such as *I^2^*, Q statistic or E-value) |  | Relevant information is presented in Supplemental tables |
| 13 | **Sensitivity analyses and additional analyses** |  |  |  |
|  | a) | Report any sensitivity analyses to assess the robustness of the main results to violations of the assumptions | 10-14 | The results of sensitivity analysis are discussed in the "Results" section |
|  | b) | Report results from other sensitivity analyses or additional analyses | 10-14 |  |
|  | c) | Report any assessment of direction of causal relationship (e.g., bidirectional MR) | 10-14 |  |
|  | d) | When relevant, report and compare with estimates from non-MR analyses | 10-14 |  |
|  | e) | Consider additional plots to visualize results (e.g., leave-one-out analyses) | 10-14 |  |
|  | **DISCUSSION** |  |  |  |
| 14 | **Key results** | Summarize key results with reference to study objectives | 15-19 | The main results are described in the "Result" section and first paragraph of the "Discussion" section. |
| 15 | **Limitations** | Discuss limitations of the study, taking into account the validity of the IV assumptions, other sources of potential bias, and imprecision. Discuss both direction and magnitude of any potential bias and any efforts to address them | 19 | The limitations of the study are described in the Second last paragraph of the "Discussion" section. |
| 16 | **Interpretation** |  |  |  |
|  | a) | Meaning: Give a cautious overall interpretation of results in the context of their limitations and in comparison with other studies | 15-19 | In conjunction with the analyzed results of this study, we have elaborated on the relevant elements in the discussion section. |
|  | b) | Mechanism: Discuss underlying biological mechanisms that could drive a potential causal relationship between the investigated exposure and the outcome, and whether the gene-environment equivalence assumption is reasonable. Use causal language carefully, clarifying that IV estimates may provide causal effects only under certain assumptions | 15-19 |  |
|  | c) | Clinical relevance: Discuss whether the results have clinical or public policy relevance, and to what extent they inform effect sizes of possible interventions | 15-19 |  |
| 17 | **Generalizability** | Discuss the generalizability of the study results (a) to other populations, (b) across other exposure periods/timings, and (c) across other levels of exposure | 19 | These results support a potential causal effect among allergic diseases. |
|  | **OTHER INFORMATION** |  |  |  |
| 18 | **Funding** | Describe sources of funding and the role of funders in the present study and, if applicable, sources of funding for the databases and original study or studies on which the present study is based | 19 | This work was supported by the Noncommunicable Chronic Diseases-National Science and Technology Major Project (2024ZD0529900), National Natural Science Foundation of China (82161138020), Major Project of Guangzhou National Laboratory (GZNL2024A02002), and Guangdong Innovation Team Project of General College and University (2023KCXTD024) |
| 19 | **Data and data sharing** | Provide the data used to perform all analyses or report where and how the data can be accessed, and reference these sources in the article. Provide the statistical code needed to reproduce the results in the article, or report whether the code is publicly accessible and if so, where | 19 | All data needed to evaluate the conclusions are presented in the paper. The resources, tools, and codes used in our analyses were described in the methods section. For any further data requests, please contact the corresponding author. |
| 20 | **Conflicts of Interest** | All authors should declare all potential conflicts of interest | 19 | The authors declare that they have no conflict of interest. |

This checklist is copyrighted by the Equator Network under the Creative Commons Attribution 3.0 Unported (CC BY 3.0) license.

1. Skrivankova VW, Richmond RC, Woolf BAR, Yarmolinsky J, Davies NM, Swanson SA, et al. Strengthening the Reporting of Observational Studies in Epidemiology using Mendelian Randomization (STROBE-MR) Statement. JAMA. 2021;under review.

2. Skrivankova VW, Richmond RC, Woolf BAR, Davies NM, Swanson SA, VanderWeele TJ, et al. Strengthening the Reporting of Observational Studies in Epidemiology using Mendelian Randomisation (STROBE-MR): Explanation and Elaboration. BMJ. 2021;375:n2233.
